# Late is too late? Surgical timing and postoperative complications after primary ileocolic resection for Crohn’s disease

**DOI:** 10.1007/s00384-022-04125-7

**Published:** 2022-03-10

**Authors:** E. Lavorini, M. E. Allaix, C. A. Ammirati, M. Astegiano, M. Morino, A. Resegotti

**Affiliations:** 1 Department of Surgical Sciences, University of Torino, C.so Dogliotti 14, 10126 Turin, Italy; 2 SC Gastroenterology U, AOU Città Della Salute E Della Scienza, Turin, Italy

**Keywords:** IBD, Crohn's disease, Surgery

## Abstract

**Background:**

Despite the recent advances in medical therapy, the majority of patients with Crohn’s disease (CD) still require surgery during the course of their life. While a correlation between early primary surgery and lower recurrence rates has been shown, the impact of surgical timing on postoperative complications is unclear. The aim of this study is to assess the impact of surgical timing on 30-day postoperative morbidity.

**Methods:**

This is a retrospective analysis of a prospectively collected database of 307 consecutive patients submitted to elective primary ileocolic resection for CD at our institution between July 1994 and July 2018. The following variables were considered: age, gender, year of treatment, smoking habits, preoperative steroid therapy, presence of fistula or abscess, type of anastomosis, and time interval between diagnosis of CD and surgery. Univariate and multivariate logistic regressions were performed to examine the association between risk factors and complications.

**Results:**

Major complications occurred in 29 patients, while anastomotic leak was observed in 16 patients. Multivariate logistic regression analysis showed that surgical timing in years (OR 1.10 *p* = 0.002 for a unit change), along with preoperative use of steroids (OR 5.45 *p* < 0.001) were independent risk factors for major complications. Moreover, preoperative treatment with steroids (6.59 *p* = 0.003) and surgical timing (OR 1.10 *p* = 0.023 for a unit change) were independently associated with anastomotic leak, while handsewn anastomosis (OR 2.84 *p* = 0.100) showed a trend.

**Conclusions:**

Our results suggest that the longer is the time interval between diagnosis of CD and surgery, the greater is the risk of major surgical complications and of anastomotic leak.

## Introduction

Crohn’s disease (CD) is a chronic and idiopathic inflammatory bowel disease (IBD) that might affect any part of the gastrointestinal tract, and for which no definitive cure is known at present. Medical treatment aims at achieving clinical remission, preventing complications, and maintaining or improving patient’s quality of life [1].

Despite recent advances in medical therapy, with an increased use of immunosuppressive and biological drugs, the majority patients with CD require surgery during the course of their life. A population-based cohort study including 1936 CD patients showed the cumulative rate of intestinal resection was 44%, 61%, and 71% at 1, 5, and 10 years after diagnosis [2].

The surgery’s main aim is to treat refractory and complicated CD and to provide long-lasting symptomatic relief. In addition, surgical resection has been shown to be an effective alternative to biologic therapy in terms of health related-quality of life [3].

However, surgery in CD patients has a high rate of complications due to many risk factors, and symptomatic recurrence rates up to 50% at 5 years have been reported [4–6].

Many factors have been found to be associated with an increased risk of complications, such as malnutrition, penetrating disease (presence of fistula or abscess), steroid, and biologic therapy.

The occurrence of postoperative complications is associated with increased risk of CD recurrence. On the other hand, there is growing evidence suggesting the association between early bowel resection and reduced postoperative recurrence rates [4, 7–9]. Based on these assumptions, we hypothesized that patients who undergo early surgery have lower postoperative morbidity than patients undergoing late surgery. To date, there are no studies specifically exploring the possible association between surgical timing and postoperative complications in CD patients. Therefore, the aim of this study was to assess whether there is a relationship between timing of surgery and postoperative morbidity.

## Methods

### Study population

This is a retrospective analysis of a prospectively collected database of consecutive patients undergoing elective primary ileocolic resection for CD at our institution between July 1994 and July 2018. Exclusion criteria were intestinal surgery other than ileocolic resection and emergent surgery.

All patients were under the care of one gastroenterologist (MA); one surgeon (AR) performed or supervised almost all ileocolic resections.

Patients’ data were collected in a predesigned Excel file. Clinical data regarding preoperative patients’ characteristics and postoperative in-hospital course were abstracted from computerized and archived patient charts if missing. The following variables were considered: age, gender, smoking habits, preoperative plasma albumin levels, preoperative steroid therapy, presence of fistula or abscess, type of anastomosis (handsewn or stapled), postoperative 30-day morbidity and mortality, and time interval between diagnosis of CD and surgery.

Postoperative complications were graded according to the Dindo-Clavien classification, considering grade I and II minor complications and grades III to V major complications [10].

### Statistical analysis

Categorical variables are presented as percentages and were compared with *X*^2^ test for multiple comparisons; continuous variables were expressed as medians and interquartile ranges (25th and 75th percentiles) and compared by the non-parametric Wilcoxon rank-sum test.

The following variables analyzed as risk factors for postoperative surgical complications (overall, minor, major and anastomotic leakage) were age, gender, year of treatment, smoking habits, preoperative plasma albumin levels, preoperative steroid therapy, presence of fistula or abscess, type of anastomosis (handsewn or stapled), and time interval between diagnosis of CD and surgery.

Multivariate logistic regression analyses were used to examine the association between different risk factors and the complication outcome after adjusting for those confounders significant at univariate analyses. The goodness of fit of the final model was checked through the Hosmer–Lemeshow test. Odds ratio (OR) and 95% confidence intervals (95%CIs) were calculated from the regression coefficients. To assess the model’s performance, the accuracy of the prediction multivariate model was expressed as area under the receiver operating characteristic (ROC) curve. Analyses were carried out using STATA statistical package 14 (Stata Corporation, College Station, TX).

## Results

Between July 1994 and June 2018, 660 patients underwent elective surgical resection for ileocolic CD. A total of 271 patients surgically treated for recurrent CD and 82 undergoing procedures other than ileocolic resection were excluded, thus leaving 307 patients for analysis. Table 1 summarizes patients’ characteristics. Of the 307 patients, 175 were males (57.0%); median age at surgery was 37 [IQR, 28–50] years, and 124 (40.4%) were smokers. Steroid treatment was used in 92 (30.0%) patients. A fistula was present at time of surgery in 154 (50.2%) patients; a total of 44 (14.3%) patients had an abscess that was not suitable for preoperative percutaneous drainage.

**Table 1:** Patients’ characteristics

	*N* = 307	Percent
Males	175	57.0
Age, median years [interq.range]	37 [28–50]	
Albumin, median, g/dl [interq range]	3.9 [3.5–4.3]	
Smoking	124	40.4
Steroids	92	30.0
Fistulas	154	50.2
Abscess	44	14.3
Year of treatment 1993–1998	71	23.1
1999–2003	63	20.5
2004–2008	61	19.9
2009–2013	57	18.6
2014–2018	55	17.9
Time interval between diagnosis of CD and surgery median years [interq.range]	4 [1–8]	
Stapled anastomosis	176	57.5
Laparoscopy	66	21.5

Median time interval between diagnosis of CD and surgery was 4 [1–8] years. Laparoscopic approach was used in 21.5% of cases.

Overall postoperative complication rate was 23.5% (*n* = 72 patients). Major complications occurred in 29 (9.4%) patients, with anastomotic leak being reported in 16 (5.2%) patients. A total of 48 (15.6%) patients experienced minor complications.

The univariate analysis for overall complications (Table 2) showed two variables to be associated with an increased risk: male gender and steroid treatment. Multivariate logistic regression analysis confirmed as independent risk factors steroid treatment [OR 2.28 (1.24–4.18) *p* = 0.008] and handsewn anastomosis [OR 0.55 (0.30–1.00) *p* = 0.050].

**Table 2:** Univariate and multivariate analyses of risk factors for overall complications

	**Surgical complications: overall complications**					
	**Univariate analyses**			**Multivariate analysis**		
	OR	95% Conf.Interval	*p*	OR	95% Conf.Interval	*p*
Males (ref. Females)	1.70	(0.98–2.96)	0.060			
Age (yrs)	1.00	(0.99–1.02)	0.652			
Albumin (g/dl)	0.93	(0.72–1.19)	0.559			
Smoking	1,13	(0.65–1.94)	0.661			
Time interval between diagnosis of CD and surgery (yrs)	1.03	(0.99–1.08)	0.124			
Steroids	1.98	(1.14–3.44)	0.015	2.28	(1.24–4.18)	0.008
Fistulas	1.23	(0.73–2.09)	0.438			
Abscess	0.82	(0.37–1.79)	0.613			
Handsewn anastomosis (ref Stapled)	0.73	(0.42–1.26)	0.255	0.55	(0.30–1.00)	0.050

**Fig. 1:**
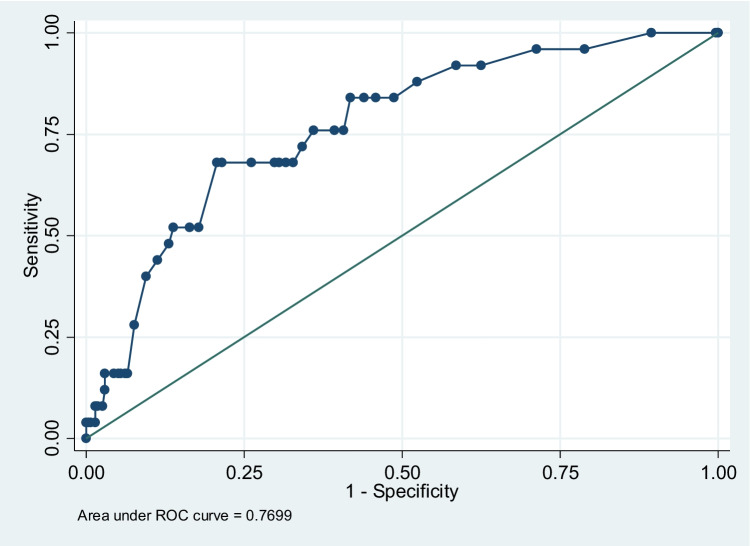
The area under the ROC curve following multivariate analysis predicting major complications

Major surgical complications (Table 3) in univariate analysis were more frequently observed in patients with a long interval between diagnosis and surgery and in those receiving medical treatment with steroids. Multivariate logistic regression analysis confirmed that surgical timing in years [OR 1.10 (1.03–1.17) *p* = 0.002 for a unit change], along with preoperative use of steroids [OR 5.45 (2.39–12.43) *p* < 0.001] were independent risk factors. The area under the ROC curve of approximately 0.77 indicates acceptable predictive power of the multivariate model (Fig. 1).

**Table 3:** Univariate and multivariate analyses of risk factors for major complications

	**Surgical complications: major complications**					
	**Univariate analyses**			**Multivariate analysis**		
	OR	95% Conf.Interval	*p*	OR	95% Conf.Interval	*p*
Males (ref. females)	1.49	(0.67–3.31)	0.333			
Age (yrs)	1.01	(0.99–1.04)	0.408			
Albumin (g/dl)	0.82	(0.39–1.72)	0.608			
Smoking	1.05	(0.47–2.37)	0.904			
Time interval between diagnosis of CD and surgery (yrs)	1.08	(1.02–1.14)	0.007	1.10	(1.03–1.17)	0.002
Steroids	4.49	(2.03–9.95)	<0.001	5.45	(2.39–12.43)	<0.001
Fistulas	1.71	(0.78–3.75)	0.182			
Abscess	0.95	(0.31–2.88)	0.931			
Handsewn anastomosis (ref stapled)	1.40	(0.64–3.04)	0.400

On univariate analysis, anastomotic leak more likely occurred in patients on steroid treatment, in those with an increased interval between diagnosis and surgery, and in those who had a handsewn anastomosis (Table 4). On multivariate analysis, preoperative treatment with steroids [6.59 (1.90–22.85) *p* = 0.003] and surgical timing [OR 1.10 (1.01–1.18) *p* = 0.023 for a unit change] were independently associated with anastomotic leak, while the construction of a handsewn anastomosis [OR 2.84 (0.82–9.87) *p* = 0.100] showed a trend toward a higher risk of anastomotic leak.

**Table 4:** Univariate and multivariate analyses of risk factors for anastomotic leak

	**Surgical complications: anastomotic leak**					
	**Univariate analyses**			**Multivariate analysis**		
	OR	95% Conf.Interval	*p*	OR	95% Conf.Interval	*p*
Males (ref. females)	1.27	(0.45–3.59)	0.649			
Age (yrs)	0.99	(0.96–1.03)	0.645			
Albumin (g/dl)	0.87	(0.38–1.99)	0.738			
Smoking	2.23	(0.77–6.44)	0.138			
Time interval between diagnosis of CD and surgery (yrs)	1.07	(1.00–1.15)	0.045	1.10	(1.01–1.18)	0.023
Steroids	7.88	(2.47–25.13)	<0.001	6.59	(1.90–22.85)	0.003
Fistulas	0.99	(0.36–2.72)	0.989			
Abscess	0.38	(0.05–2.99)	0.361			
Handsewn anastomosis (ref stapled)	4.37	(1.38–13.89)	0.012	2.84	(0.82–9.87)	0.100

## Discussion

CD is a condition that is primarily managed with medical treatment. Surgery plays a major role in the treatment of complicated and refractory CD. However, surgery is not curative: almost 70% of patients develop new lesions at the neoterminal ileum (endoscopic recurrence) at 1 year after ileo-cecal resection, 28–50% of patients develop symptoms (clinical recurrence) after 5 years, and a quarter of patients will need further bowel resection for complications or refractory disease (surgical recurrence) [6, 11].

According to Yamamoto et al., rates of postoperative morbidity after CD surgery are up to 24% [12]. Main risk factors for postoperative complications in patients undergoing primary surgery for CD include preoperative use of steroids, intra-abdominal abscess or fistula, malnutrition, and positive histological inflammatory margins on the specimen [13–23].

Some authors have found an association between postoperative complications and early CD recurrence. For instance, Iesalnieks et al. [24] found that patients with postoperative intra-abdominal septic complications had significantly higher surgical recurrence rate than patients without postoperative complications. Similarly, a recent US study from the Cleveland Clinic [25] found a strong correlation (OR 12.1) between postoperative complications and recurrence. Lastly, Kanazawa et al. [21] reported that the reoperation rate at 1 year was 41.2% in those patients who experienced complications and 2.3% in those without complications.

Based on these data suggesting higher recurrence rates in patients with postoperative morbidity, surgery is postponed as far as possible. However, there is growing evidence suggesting that early bowel resection is associated with reduced postoperative recurrence rates [7–9].

Having in mind that early surgery might reduce postoperative CD recurrence and considering the existing correlation between postoperative morbidity and recurrence, we decided to investigate if early surgery may lead to lower postoperative morbidity. Our hypothesis was that patients who do not benefit from medical therapy and undergo early surgery have significantly reduced postoperative morbidity than patients undergoing late surgery.

To our best knowledge, no studies have specifically explored possible associations between surgical timing and postoperative complications in CD patients. To date, the concept of early or late surgery is controversial and debated in the literature: “late” surgery is more likely considered that is performed to rescue patients failing medical treatment [26], while “early” surgery mainly refers to the procedure performed in those patients with acute complication as first presentation of CD [9].

On the contrary, we herein consider early and late surgical timing just referring to the time span between the diagnosis of CD and surgery, regardless of the presence of CD-related complications. We agree with the universally accepted concept that main indications for surgery are complicated and refractory CD. However, the prompt identification of those CD patients who will need surgery during their life might lower the risk of surgery-related morbidity. The results of our study including 307 patients undergoing primary surgery for ileocolic CD show that the longer is the time interval between diagnosis of CD and surgery, the greater is the risk of major surgical complications and of anastomotic leak.

Currently, one of the main challenges in the treatment of CD patients is the lack of early predictors of the development of aggressive CD, and therefore the impossibility of identifying those patients who may benefit from early surgery. Short un-complicated ileocaecal CD is one of the few recognized and well-established indications for surgery at front [27].

Otherwise, the definition of the correct surgical timing is more complex and is mainly achieved by the strict cooperation between expert gastroenterologists and surgeons.

Our study also identified steroid therapy as a strong independent risk factor for major postoperative morbidity and for anastomotic dehiscence, confirming the data from previous studies. This can be interpreted in two ways: increased morbidity may be a specific drug side effect on anastomotic healing or it may reflect disease activity.

Perforating CD is a known risk factor for postoperative morbidity [13, 14, 18]. However, the results of this study failed to demonstrate a significant relationship between the presence of a fistula or abscess and major postoperative complications. This might be due to the fact that 30.9% of patients with perforating CD had a covering ileostomy at index surgery, as compared to 13.6% with no perforating CD.

The impact of the anastomotic technique on anastomotic leak is controversial. Some authors have found that a stapled side-to-side anastomosis has reduced leak rates as compared to handsewn end-to-end anastomosis [28–33], while others have reported no difference between stapled side-to-side and handsewn end-to-end anastomosis [34, 35]. In our study, the handsewn anastomosis showed a trend toward a higher rate of anastomotic leaks, even though a statistical significance was not reached.

We acknowledge that this study has some limitations. First, it is retrospective in nature, over a 25-year period. Such time span might be considered too long; however, the indications for surgery did not change over time and the vast majority of patients were treated by one dedicated gastroenterologist and one surgeon devoted to IBD surgery. In addition, even though one might speculate that the improvements in medical therapy observed in the last 20 years may have an impact on postoperative complications, there is no evidence in the literature suggesting a significant reduction in early postoperative morbidity over time. Second, it is a single-institution study; as a consequence, the results may be not generalized.

In conclusion, our results suggest that early surgery in those CD patients who will need surgery during their life might reduce postoperative morbidity. Further studies are required to better select this subgroup of patients. At present, the right timing for surgery is best defined by a dedicated multidisciplinary team in an IBD unit.
